# Supplementary material for: Smooth Interpolating Curves with Local Control and Monotone Alternating Curvature
Source: Comput Graph Forum. 2022 Oct 6;41(5):25–38. doi: 10.1111/cgf.14600 (PMC9827861; doi:10.1111/cgf.14600)
Supplement: Supplementary file 1 — Supplement Material [file CGF-41-25-s001.zip › Local-Smooth-Interpolating-MonoCurvature/extern/clothoids/docs/api-cpp/program_listing_file_Triangle2D.cc.html]

Program Listing for File Triangle2D.cc — Clothoids v2.0.9

### Navigation

- index
- toc
- Clothoids »
- Program Listing for File Triangle2D.cc

# Program Listing for File Triangle2D.cc¶

↰ Return to documentation for file (`Triangle2D.cc`)

```
/*
 *  Triangle-Triangle Overlap Test Routines
 *  July, 2002
 *  Updated December 2003
 *
 *  This file contains C implementation of algorithms for
 *  performing two and three-dimensional triangle-triangle intersection test
 *  The algorithms and underlying theory are described in
 *
 *  "Fast and Robust Triangle-Triangle Overlap Test Using Orientation Predicates"
 *  P. Guigue - O. Devillers
 *
 *  Journal of Graphics Tools, 8(1), 2003
 *
 *  Several geometric predicates are defined.  Their parameters are all
 *  points.  Each point is an array of two or three double precision
 *  floating point numbers. The geometric predicates implemented in
 *  this file are:
 *
 *    int tri_tri_overlap_test_3d(p1,q1,r1,p2,q2,r2)
 *    int tri_tri_overlap_test_2d(p1,q1,r1,p2,q2,r2)
 *
 *    int tri_tri_intersection_test_3d(p1,q1,r1,p2,q2,r2,
 *                                     coplanar,source,target)
 *
 *       is a version that computes the segment of intersection when
 *       the triangles overlap (and are not coplanar)
 *
 *    each function returns 1 if the triangles (including their
 *    boundary) intersect, otherwise 0
 *
 *
 *  Other information are available from the Web page
 *  http://www.acm.org/jgt/papers/GuigueDevillers03/
 *
 */
/*
 *
 *  Two dimensional Triangle-Triangle Overlap Test
 *
 */

#include "Clothoids.hh"

// workaround for windows that defines max and min as macros!
#ifdef max
  #undef max
#endif
#ifdef min
  #undef min
#endif

#include <functional>
#include <algorithm>

namespace G2lib {

  using std::min;
  using std::max;
  using std::swap;

  #ifndef DOXYGEN_SHOULD_SKIP_THIS

  static
  inline
  real_type
  orient_2d(
    real_type const a[2],
    real_type const b[2],
    real_type const c[2]
  ) {
    return (a[0]-c[0]) * (b[1]-c[1]) - (a[1]-c[1]) * (b[0]-c[0]);
  }

  static
  inline
  bool
  intersection_test_vertex(
    real_type const P1[2],
    real_type const Q1[2],
    real_type const R1[2],
    // - - - - - - - - - -
    real_type const P2[2],
    real_type const Q2[2],
    real_type const R2[2]
  ) {
    if ( orient_2d(R2,P2,Q1) >= 0 ) {
      if ( orient_2d(R2,Q2,Q1) <= 0 ) {
        if ( orient_2d(P1,P2,Q1) > 0 ) {
          return orient_2d(P1,Q2,Q1) <= 0;
        } else {
          return orient_2d(P1,P2,R1) >= 0 && orient_2d(Q1,R1,P2) >= 0;
        }
      } else {
        return orient_2d(P1,Q2,Q1) <= 0 &&
               orient_2d(R2,Q2,R1) <= 0 &&
               orient_2d(Q1,R1,Q2) >= 0;
      }
    } else {
      if ( orient_2d(R2,P2,R1) >= 0 ) {
        if ( orient_2d(Q1,R1,R2) >= 0 ) {
          return orient_2d(P1,P2,R1) >= 0;
        } else {
          return orient_2d(Q1,R1,Q2) >= 0 && orient_2d(R2,R1,Q2) >= 0;
        }
      } else {
        return false;
      }
    }
  }

  // - - - - - - - - - - - - - - - - - - - - - - - - - - - - - - - - - - - - - -

  static
  inline
  bool
  intersection_test_edge(
    real_type const P1[2],
    real_type const Q1[2],
    real_type const R1[2],
    real_type const P2[2],
    real_type const R2[2]
  ) {
    if ( orient_2d(R2,P2,Q1) >= 0 ) {
      if ( orient_2d(P1,P2,Q1) >= 0 ) {
        return orient_2d(P1,Q1,R2) >= 0;
      } else {
        return orient_2d(Q1,R1,P2) >= 0 && orient_2d(R1,P1,P2) >= 0;
      }
    } else if ( orient_2d(R2,P2,R1) >= 0 ) {
      return orient_2d(P1,P2,R1) >= 0 &&
             ( orient_2d(P1,R1,R2) >= 0 || orient_2d(Q1,R1,R2) >= 0 );
    }
    return false;
  }

  // - - - - - - - - - - - - - - - - - - - - - - - - - - - - - - - - - - - - - -

  static
  inline
  bool
  tri_tri_intersection_2d(
    real_type const p1[2],
    real_type const q1[2],
    real_type const r1[2],
    real_type const p2[2],
    real_type const q2[2],
    real_type const r2[2]
  ) {
    if ( orient_2d(p2,q2,p1) >= 0 ) {
      if ( orient_2d(q2,r2,p1) >= 0 ) {
        return orient_2d(r2,p2,p1) >= 0 || intersection_test_edge(p1,q1,r1,p2,r2);
      } else {
        if ( orient_2d(r2,p2,p1) >= 0 ) return intersection_test_edge(p1,q1,r1,r2,q2);
        else                            return intersection_test_vertex(p1,q1,r1,p2,q2,r2);
      }
    } else {
      if ( orient_2d(q2,r2,p1) >= 0 ) {
        if ( orient_2d(r2,p2,p1) >= 0 ) return intersection_test_edge(p1,q1,r1,q2,p2);
        else                            return intersection_test_vertex(p1,q1,r1,q2,r2,p2);
      } else {
        return intersection_test_vertex(p1,q1,r1,r2,p2,q2);
      }
    }
  }

  // - - - - - - - - - - - - - - - - - - - - - - - - - - - - - - - - - - - - - -

  static
  inline
  bool
  tri_tri_overlap_test_2d(
    real_type const p1[2],
    real_type const q1[2],
    real_type const r1[2],
    real_type const p2[2],
    real_type const q2[2],
    real_type const r2[2]
  ) {
    if ( orient_2d(p1,q1,r1) < 0 ) {
      if ( orient_2d(p2,q2,r2) < 0 )
        return tri_tri_intersection_2d(p1,r1,q1,p2,r2,q2);
      else
        return tri_tri_intersection_2d(p1,r1,q1,p2,q2,r2);
    } else {
      if ( orient_2d(p2,q2,r2) < 0 )
        return tri_tri_intersection_2d(p1,q1,r1,p2,r2,q2);
      else
        return tri_tri_intersection_2d(p1,q1,r1,p2,q2,r2);
    }
  }

  #endif

  // - - - - - - - - - - - - - - - - - - - - - - - - - - - - - - - - - - - - - -

  void
  Triangle2D::rotate( real_type angle, real_type cx, real_type cy ) {
    real_type C   = cos(angle);
    real_type S   = sin(angle);

    real_type dx  = m_p1[0] - cx;
    real_type dy  = m_p1[1] - cy;
    real_type ndx = C*dx - S*dy;
    real_type ndy = C*dy + S*dx;
    m_p1[0] = cx + ndx;
    m_p1[1] = cy + ndy;

    dx      = m_p2[0] - cx;
    dy      = m_p2[1] - cy;
    ndx     = C*dx - S*dy;
    ndy     = C*dy + S*dx;
    m_p2[0] = cx + ndx;
    m_p2[1] = cy + ndy;

    dx      = m_p3[0] - cx;
    dy      = m_p3[1] - cy;
    ndx     = C*dx - S*dy;
    ndy     = C*dy + S*dx;
    m_p3[0] = cx + ndx;
    m_p3[1] = cy + ndy;

  }

  // - - - - - - - - - - - - - - - - - - - - - - - - - - - - - - - - - - - - - -

  bool
  Triangle2D::overlap( Triangle2D const & t2 ) const {
    return tri_tri_overlap_test_2d( m_p1, m_p2, m_p3, t2.m_p1, t2.m_p2, t2.m_p3 );
  }

  // - - - - - - - - - - - - - - - - - - - - - - - - - - - - - - - - - - - - - -

  real_type
  Triangle2D::distMax( real_type x, real_type y ) const {
    real_type d1 = hypot( x-m_p1[0], y-m_p1[1] );
    real_type d2 = hypot( x-m_p2[0], y-m_p2[1] );
    real_type d3 = hypot( x-m_p3[0], y-m_p3[1] );
    return max(d1,max(d2,d3));
  }

  // - - - - - - - - - - - - - - - - - - - - - - - - - - - - - - - - - - - - - -

  #ifndef DOXYGEN_SHOULD_SKIP_THIS

  static
  real_type
  distSeg(
    real_type         x,
    real_type         y,
    real_type const * A,
    real_type const * B
  ) {
    real_type dx  = x    - A[0];
    real_type dy  = y    - A[1];
    real_type dx1 = B[0] - A[0];
    real_type dy1 = B[1] - A[1];

    // < P-A - s*(B-A), B-A> = 0
    // <P-A, B-A> = s <B-A,B-A>

    real_type tmp = dx * dx1 + dy * dy1;

    if ( tmp < 0 ) return hypot(dx,dy);

    real_type tmp2 = dx1*dx1+dy1*dy1;

    if ( tmp > tmp2 ) return hypot(x-B[0],y-B[1]);

    real_type S = tmp/tmp2;
    real_type X = A[0] + S*dx1;
    real_type Y = A[1] + S*dy1;

    return hypot( x-X, y-Y );
  }

  #endif

  real_type
  Triangle2D::distMin( real_type x, real_type y ) const {

    int_type in = isInside( x, y );
    if ( in >= 0 ) return 0;

#if 0
    LineSegment L1, L2, L3;
    L1.build_2P( p1, p2 );
    L2.build_2P( p2, p3 );
    L3.build_2P( p3, p1 );

    real_type d1 = L1.distance( x, y );
    real_type d2 = L2.distance( x, y );
    real_type d3 = L3.distance( x, y );
#else
    real_type d1 = distSeg( x, y, m_p1, m_p2 );
    real_type d2 = distSeg( x, y, m_p2, m_p3 );
    real_type d3 = distSeg( x, y, m_p3, m_p1 );
#endif

    if ( d1 > d2 ) swap( d1, d2 );
    if ( d1 > d3 ) swap( d1, d3 );
    return d1;

  }

  // - - - - - - - - - - - - - - - - - - - - - - - - - - - - - - - - - - - - - -

  ostream_type &
  operator << ( ostream_type & stream, Triangle2D const & t ) {
    fmt::print( stream,
      "Triangle2D\n"
      "P0 = [{},{}]\n"
      "P1 = [{},{}]\n"
      "P2 = [{},{}]\n",
      t.m_p1[0], t.m_p1[1],
      t.m_p2[0], t.m_p2[1],
      t.m_p3[0], t.m_p3[1]
    );
    return stream;
  }

}
```

### Quick search

### Table of Contents

- Matlab Interface Manual
- C++ API
- MATLAB API

«
hide menu

menu
sidebar
»

### Navigation

- index
- toc
- Clothoids »
- Program Listing for File Triangle2D.cc

© Copyright 2021, Enrico Bertolazzi and Marco Frego.
Created using Sphinx 4.2.0.
